# Treatment Advances in Gynecologic Cancers: Insights From Recent Clinical Trials

**DOI:** 10.1002/cai2.70065

**Published:** 2026-06-07

**Authors:** Yiyang Shen, Xiaofei Jiao, Yuanjia Wen, Zikun Peng, Xuejiao Zhao, Jiahao Liu, Qinglei Gao

**Affiliations:** ^1^ Department of Gynecological Oncology, Tongji Hospital, Tongji Medical College Huazhong University of Science and Technology Wuhan Hubei China; ^2^ National Clinical Research Center for Obstetrics and Gynecology, Key Laboratory of Cancer Invasion and Metastasis (Ministry of Education), Hubei Key Laboratory of Tumor Invasion and Metastasis, Tongji Hospital, Tongji Medical College Huazhong University of Science and Technology Wuhan Hubei China; ^3^ Division of Oncological Sciences Knight Cancer Institute Oregon Health and Science University Portland Oregon USA

**Keywords:** antibody‐drug conjugates, clinical trials, gynecologic cancers, immunotherapy, treatment advances

## Abstract

Gynecologic cancers remain a major global health burden for women. Their management has evolved toward a gynecologic oncologist‐led multimodal paradigm. While these approaches have improved outcomes, critical limitations persist, including challenges in decision‐making during surgical procedures and suboptimal efficacy coupled with frequent resistance and substantial toxicities during medical management. Recent high‐quality clinical trials have helped refine these areas. In early‐stage cervical cancer, sentinel lymph node biopsy has emerged as a safe and effective alternative to systematic lymphadenectomy, preserving oncologic outcomes while minimizing morbidity. In advanced ovarian cancer, primary debulking surgery has been established as superior to neoadjuvant chemotherapy followed by interval debulking surgery in fit patients treated at experienced centers. Meanwhile, immunotherapy demonstrates promise through strategic combinations, including dual checkpoint inhibition and triplet regimens incorporating chemotherapy and anti‐angiogenic agents. Antibody‐drug conjugates (ADCs) directed against folate receptor alpha (FRα) and tissue factor (TF) have shown durable responses in biomarker‐selected populations. By critically synthesizing these advances, this review seeks to delineate the clinical utility of emerging therapies, highlight knowledge gaps, and inform evidence‐based decision‐making for clinicians and researchers.

## Graphical abstract

Overview of immunotherapy and antibody‐drug conjugates, and their mechanism against cancer. (The Graphical Abstract Image was created with BioRender.)

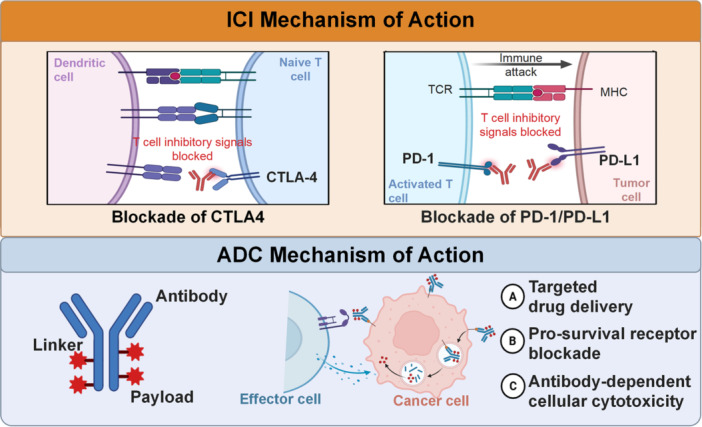

**Abbreviations**
ADC: antibody‐drug conjugate
CI: confidence interval
CTLA‐4: cytotoxic T‐lymphocyte‐associated protein 4
dMMR: deficient mismatch repair
FRα: folate receptor alpha
HER2: human epidermal growth factor receptor 2
HR: hazard ratio
ICI: immune checkpoint inhibitor
IDS: interval debulking surgery
NACT: neoadjuvant chemotherapy
ORR: objective response rate
OS: overall survival
PARP: poly(ADP‐ribose) polymerase
PDS: primary debulking surgery
PD‐1: Programmed Death‐1
PD‐L1: Programmed Death‐Ligand 1
PFS: progression‐free survival
pMMR: proficient mismatch repair
SLN: sentinel lymph node
TF: tissue factor
TROP2: trophoblast cell surface antigen 2

## 1 Background

Gynecologic cancers, primarily involving malignancies of the cervix, ovary, and endometrium, constitute a substantial global health burden for women [1, 2]. Among these diseases, cervical cancer exhibits the highest incidence, accounting for approximately 14% of all female cancers worldwide and resulting in over 300,000 deaths annually [3]. It remains the predominant type and the leading cause of cancer‐related mortality among gynecologic cancers in most developing nations [4]. Epithelial ovarian cancer, the most common subtype of ovarian malignancy, is marked by late‐stage diagnosis, frequent development of drug resistance, and inevitable recurrence, making it the most fatal form of gynecologic cancers, with a 5‐year survival rate of less than 50% [5]. As for endometrial cancer, while it generally presents a favorable prognosis, its incidence is rising rapidly, attributed to improving socioeconomic conditions and lifestyle factors, such as obesity and hormonal imbalances, with an annual increase of 1%–2% in developed regions [6, 7].

Traditional management of gynecologic cancers has long relied on a multimodal approach managed by gynecologic oncologists and integrating surgery, chemotherapy, and radiotherapy [8]. Surgery remains the cornerstone of treatment. Radical hysterectomy is the standard procedure for early‐stage cervical cancer [3], whereas cytoreductive surgery plays a central role in ovarian cancer management, with the goal of achieving complete or optimal cytoreduction [9]. For advanced disease, systemic chemotherapy is often incorporated into multidisciplinary management, with platinum‐based regimens and paclitaxel remaining central agents [10]. In selected patients with advanced gynecologic malignancies, neoadjuvant platinum–taxane chemotherapy may be considered when upfront surgery is not feasible or when complete cytoreduction is unlikely to be achieved [11]. Radiotherapy also plays an important role in cervical cancer, particularly in the form of external beam radiotherapy combined with brachytherapy for local control [12, 13].

Despite these advances, important challenges remain [14]. Surgical decision‐making remains contentious, particularly regarding optimal timing, technique, and resection extent for heterogeneous patient populations [15]. Chemotherapy and radiotherapy, while effective, are hampered by high recurrence rates, unavoidable resistance, and substantial toxicities [16, 17]. Targeted therapies such as PARP inhibitors and anti‐angiogenics have partially addressed these gaps, yet their benefit is still constrained by biomarker heterogeneity, evolving resistance, and imperfect patient selection [17, 18]. At the same time, novel treatments, such as immune checkpoint inhibitors (ICIs) and antibody‐drug conjugates (ADCs), have generated increasingly robust clinical trial evidence in gynecologic oncology [19, 20].

Addressing these unmet needs is essential for advancing precision medicine in gynecologic oncology. Recent clinical trials have provided important evidence to refine surgical practice, optimize systemic therapy, and improve patient selection. In this review, we summarize recent advances in both surgical and medical treatment, highlight their implications for clinical practice, and discuss the key questions that remain unresolved.

## 2 Refinement of Surgical Procedures

Surgical resection remains the cornerstone of treatment for most gynecologic cancers. Standardized surgical protocols, established over decades, are widely adopted in clinical practice. In early‐stage cervical and endometrial cancers, which are often curable with surgery, current debate focuses on whether the extent of resection can be safely reduced without compromising oncologic outcomes. In ovarian cancer, where surgery is primarily aimed to reduce tumor burden and prolong survival, the primary controversy revolves around the optimal timing of cytoreductive surgery. Recent trials have provided important data on both issues.

### 2.1 Preserving Tumor‐Draining Lymph Nodes in Cervical Cancer

Since the establishment of the Wertheim procedure over a century ago, systematic pelvic lymphadenectomy has remained the unshakeable standard for surgical staging in cervical cancer [21]. However, this one‐size‐fits‐all approach faces a fundamental clinical paradox: while lymph node status is decisive for prognosis, nodal metastasis occurs in only a small fraction (approximately 10%–15%) of early‐stage patients. Consequently, the vast majority of women are exposed to the lifelong morbidity of systematic clearance, such as lymphedema and lymphocysts [22], without therapeutic benefit. As surgical philosophy evolves from maximum radicality to “precision preservation,” the concept of “less is more” has become the guiding principle for modern oncology. Although sentinel‐lymph‐node (SLN) biopsy has matured as a technique with proven diagnostic accuracy [23, 24, 25], wider adoption has been limited by the lack of definitive comparative survival data [26]. The PHENIX trial was designed to address this evidence gap [27].

The PHENIX trial provides high‐quality answers through a pragmatic yet rigorous design, specifically targeting patients with FIGO 2009 stage IA1 (LVSI+) to IIA1 disease and a tumor diameter ≤ 3 cm. Its pivotal methodological innovation was intraoperative randomization: only patients with frozen‐section‐negative sentinel nodes were randomized, thereby restricting the comparison to an intraoperatively defined low‐risk population. With a median follow‐up of 62.8 months, the study met its primary endpoint robustly. The 3‐year disease‐free survival was 96.9% in the biopsy‐only group versus 94.6% in the lymphadenectomy group (difference, −2.3 percentage points; 95% confidence interval [CI], −5.0 to 0.5), conclusively demonstrating non‐inferiority. The clinical rationale for de‐escalation was further cemented by the safety data. The biopsy‐only strategy profoundly reduced long‐term sequelae, including lower‐extremity lymphedema (5.2% vs. 19.1%) and lymphocysts (8.3% vs. 22.0%). Crucially, the trial sheds new light on the ongoing controversy surrounding minimally invasive surgery. In the laparoscopic subgroup, patients assigned to SLN biopsy alone achieved excellent outcomes (3‐year disease‐free survival of 97.3%), whereas those undergoing laparoscopic systemic lymphadenectomy performed worse (93.6%). This finding suggests that the inferior outcomes observed in previous studies (e.g., the LACC trial) might be partly attributable to the tumor dissemination risks associated with extensive radical dissection under pneumoperitoneum, rather than the laparoscopic approach itself [28]. By adhering to the “tumor‐free principle” and minimizing dissection scope, SLN biopsy may offer a safe pathway for minimally invasive surgery in this setting [29, 30].

PHENIX marks a paradigm shift in cervical cancer surgery from anatomical radicality toward a more precise and less morbid approach [31, 32]. It proves that for appropriately selected patients, oncologic safety does not necessarily require systematic lymphadenectomy, and that preservation of normal lymphatic drainage may translate into better overall outcomes. Moving forward, implementation must focus on strict adherence to these selection criteria, high‐quality tracer mapping, and standardized pathologic assessment, including ultrastaging where feasible. The optimal management of SLN‐positive patients remains to be clarified, and the ongoing PHENIX‐II cohort, together with future translational research, may help address this question. Overall, PHENIX symbolizes the future of surgery: a future defined not just by the sharpness of the scalpel, but by the power of evidence to deliver patient‐centered care.

### 2.2 Time of Debulking Surgery in Ovarian Cancer

The optimal management strategy for patients with newly diagnosed advanced ovarian cancer remains a longstanding area of debate. Standard care has consisted of primary debulking surgery (PDS) followed by systemic chemotherapy. In recent years, neoadjuvant chemotherapy (NACT) followed by interval debulking surgery (IDS) has been increasingly recommended for patients who are unlikely to achieve optimal cytoreduction through PDS or who are unfit to tolerate aggressive primary surgical intervention due to poor condition [1]. Based on evidence from several phase III randomized trials comparing NACT versus conventional treatment, including EORTC 55971 [33], CHORUS [34], SCORPION [35], and JCOG0602 [36], NACT‐IDS has been demonstrated to increase complete resection rate and reduce perioperative morbidity. However, it has not shown survival benefits and may be associated with a potential risk of shortening progression‐free survival (PFS) and/or overall survival (OS) in patients with less extensive diseases [37]. Even though PFS and OS were comparable between the two arms in certain studies, the survival outcomes were notably inferior compared with those reported in other studies of conventional treatment. These suboptimal survival outcomes may be attributable to patient selection bias and surgical quality disparity [38]. Therefore, the debate regarding the selection and identification of optimal surgical candidates, as well as the timing of surgical intervention, remains unresolved.

The trial on radical upfront surgical therapy (TRUST), a phase III, multi‐center study, was designed and started in 2016 to revisit this question under strict surgical quality control [38]. The enrolled participants were FIGO stage IIIB–IVB epithelial ovarian cancer patients with good performance status (Eastern Cooperative Oncology Group 0/1) to tolerate the procedures required to attain complete tumor resection. The most noteworthy aspect of this trial, compared with previous research, is its exceptionally stringent control over surgical quality and surgical scope. Participating centers were required to adhere to a rigorous onsite surgical quality assurance audit and demonstrate adequate infrastructure, surgical proficiency (a complete resection rate of at least 50% in PDS), and sufficient procedural volume (averaging at least 36 PDS per year). Preliminary data presented at the 2025 ASCO annual meeting revealed a significant PFS benefit for PDS over NACT‐IDS in this highly selected population [39]. Among 688 randomized patients, median PFS was 22.2 months with PDS and 19.7 months with NACT‐IDS (*p* = 0.02). Median OS numerically favored PDS (54.3 vs. 48.3 months), although this difference did not reach statistical significance (*p* = 0.24). As in previous studies, the surgical outcomes in the IDS group were superior to those in the PDS group, with R0 resection rates of 76.6% and 62.9%, respectively.

These findings highlight an important clinical paradox: a higher R0 resection rate after NACT does not necessarily translate into better long‐term survival. One possible explanation is that preoperative chemotherapy may alter tumor biology, enrich for resistant clones, or make microscopic residual disease more difficult to identify and eradicate [40]. The findings from the TRUST trial indicate that, even with high‐quality surgical assurance, merely increasing the R0 resection rate through NACT, while improving surgical outcomes, is insufficient to fundamentally alter the biological trajectory of the disease. Future therapeutic strategies should focus on fundamental breakthroughs rather than just optimizing local treatments to significantly enhance long‐term patient survival. On the one hand, it is crucial to implement precise patient stratification based on molecular profiling of tumors, enabling the integration of surgery and chemotherapy with personalized therapies, such as targeted and immunotherapeutic approaches, thereby achieving precision medicine. On the other hand, advancing the understanding of the mechanisms underlying disease initiation, metastasis, and therapeutic resistance through rigorous basic experimental research provides a critical scientific foundation for the development of novel, radical interventional approaches.

An unignorable consideration is whether the TRUST results can be generalized beyond high‐volume, expert surgical centers. The observed benefits in PFS with PDS may be difficult to replicate without targeted investments in training, centralized care, and quality monitoring. Health systems with robust referral networks and skilled surgical programs may adopt TRUST‐based recommendations more easily. However, in resource‐limited or community settings, improving surgical quality, standardizing protocols, and exploring alternative strategies such as NACT‐IDS optimization will be necessary for effective implementation.

Collectively, the TRUST trial mitigated the constraints of earlier investigations by implementing rigorous central qualification review and surgical standardization, providing high‐quality evidence and the first definitive demonstration of the superiority of PDS over IDS in terms of long‐term survival, further solidifying PDS as the standard‐of‐care treatment. More detailed data are pending further updates and publication.

### 2.3 New Perspectives for Traditional Surgeries

The PHENIX and TRUST trials, though focused on distinct gynecologic cancers, converged on a shared focus: redefining the role of traditional surgical oncology through evidence‐based innovation. PHENIX supports surgical de‐escalation in carefully selected early‐stage cervical cancer, whereas TRUST reinforces the importance of upfront high‐quality cytoreduction in appropriately selected ovarian cancer. Collectively, these findings underscore that surgical outcomes are contingent upon tumor biology, treatment sequencing, and rigorous quality control—rather than procedural intensity alone. The future of gynecologic surgery lies in shifting the paradigm from “more is better” to “right is better,” prioritizing selectively targeted, evidence‐driven strategies that balance efficacy with patient‐centered outcomes.

## 3 Advances in Medical Treatment

As precision medicine evolves, recent advances in the medical management of gynecologic cancers have focused on immunotherapy and ADCs. In the immunotherapy field, the most important progress has come from rational combination strategies and biomarker‐driven personalized applications. For ADCs, studies have validated their capacity to enable precise delivery of cytotoxic regimens to cancer cells while minimizing off‐target toxicity to healthy tissues.

### 3.1 Advanced Immunotherapy

**Figure 1:**
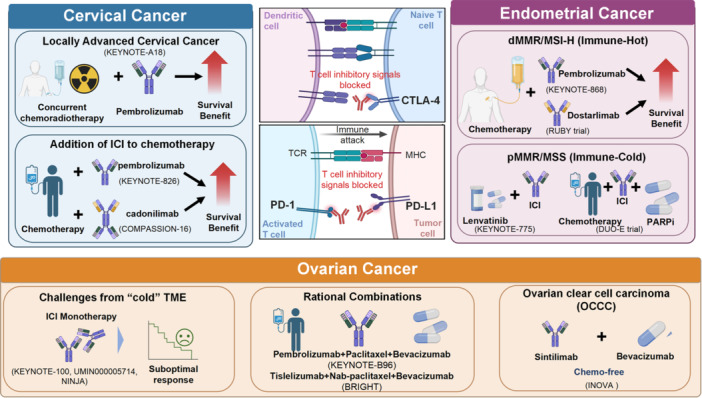
Overview of advances in immunotherapy for cervical cancer, endometrial cancer, and ovarian cancer. Created in BioRender. Liu, J. (2026) https://BioRender.com/ciehj17.

Immunotherapy is based on the premise that endogenous antitumor immunity can be restored or amplified even within an immunosuppressive tumor microenvironments. This principle underpins the clinical success of ICIs, exemplified by agents targeting programmed cell death protein 1 (PD‐1)/PD‐ligand 1 (PD‐L1) and cytotoxic T‐lymphocyte‐associated antigen 4 (CTLA‐4). Notably, ICI‐mediated immune activation not only eradicates established tumors but also establishes sustained antitumor immunity, offering the tantalizing prospect of durable remission or even cure. However, despite its theoretical promise, critical gaps remain in defining optimal therapeutic strategies, identifying biomarkers for patient stratification, and elucidating mechanisms of resistance (Figure 1).

#### 3.1.1 ICI in Cervical Cancer

For locally advanced cervical cancer (LACC), ICI has been evaluated in combination with chemoradiotherapy. In the phase III trial KEYNOTE‐A18 (ENGOT‐cx11/GOG‐3047), pembrolizumab given concurrently with standard concurrent chemoradiotherapy (CCRT) and continued as maintenance significantly improved PFS (hazard ratio [HR] = 0.72; 95% CI 0.59–0.87) and OS (HR = 0.73; 95% CI 0.57–0.94) compared to CCRT alone [41, 42]. These data mark the first time in over two decades that immunologic priming during radiation combined with sustained checkpoint inhibition can improve long‐term disease control in high‐risk LACC, establishing a new global standard of care for high‐risk LACC [42]. However, not all radiotherapy approaches have succeeded in unselected, all‐comer populations. In CALLA, durvalumab given with and following chemoradiotherapy did not significantly improve PFS (HR = 0.84, 95% CI 0.65–1.08; *p* = 0.17) [43]. Taken together, these divergent results emphasize the importance of treatment design and potentially patient selection in this setting, and highlight the need for biomarker‐guided refinement to maximize benefit in LACC.

In persistent, recurrent, or metastatic cervical cancer, several phase III trials now establish both single‐target and dual‐target checkpoint strategies as clinically effective. KEYNOTE‐826 proved the favorable prognosis of adding pembrolizumab (anti‐PD‐1) to platinum‐based chemotherapy, with optional bevacizumab [44, 45]. The BEATcc trial further showed that adding atezolizumab (anti‐PD‐L1) to bevacizumab plus platinum‐based chemotherapy improved both PFS and OS [46]. More recently, COMPASSION‐16 evaluated cadonilimab, a bispecific antibody targeting PD‐1/CTLA‐4, combined with platinum‐based chemotherapy with or without bevacizumab, and reported significant improvements in PFS (12.7 vs. 8.1 months; HR = 0.62, 95% CI 0.49–0.80) and OS (not reached vs. 22.8 months; HR = 0.64, 95% CI 0.48–0.86) [47]. These results support both single blockade of PD‐1 and dual blockade of PD‐1/CTLA‐4 as the first‐line standard backbone in this setting. Future studies are poised to expand on these findings, seeking to determine the best combination approaches to potentiate immune responses in recurrent or metastatic disease.

#### 3.1.2 ICI in Endometrial Cancer

Endometrial cancer is notable for having one of the highest proportions of mismatch repair‐deficient (dMMR) or microsatellite instability‐high (MSI‐H) tumors among common solid malignancies. Accordingly, multiple phase III trials, including NRG‐GY018/KEYNOTE‐868 [48, 49] and RUBY [50], have consistently demonstrated substantial benefit from adding immune checkpoint blockade to first‐line carboplatin/paclitaxel in the dMMR subgroup. Clinically, however, the larger challenge remains improving outcomes in the more common proficient MMR (pMMR) population and in patients with recurrent disease.

For pMMR tumors, which lack the high neoantigen load compared to dMMR tumors, single‐agent checkpoint inhibition is often insufficient. Current research focuses on novel combinations to enhance immunogenicity in pMMR endometrial cancer patients. DUO‐E tested carboplatin/paclitaxel plus durvalumab followed by maintenance durvalumab, with or without olaparib. Notably, the addition of olaparib in the maintenance phase provided a PFS benefit specifically in the pMMR population (HR = 0.57 vs. the control group; HR = 0.76 vs. durvalumab alone group) [51]. These results support the rationale that targeted inhibition of DNA damage response, like PARP inhibitors, may reinforce immune‐mediated long‐term disease control in pMMR settings. In previously treated advanced/recurrent endometrial cancer, study 309/KEYNOTE‐775 showed that lenvatinib (targeting VEGFR/FGFR) plus pembrolizumab significantly improved PFS and OS versus chemotherapy in both the pMMR cohort and the overall population cohort [52, 53]. These data support that combining checkpoint blockade with agents that enhance tumor immunogenicity or modify the tumor microenvironment through targeting DNA damage response or the VEGF pathway can improve outcomes when PD‐1 monotherapy is inadequate.

#### 3.1.3 ICI in Ovarian Cancer

Ovarian cancer presents unique challenges for immunotherapy [54]. Despite over 75% of patients responding to platinum‐based first‐line chemotherapy, relapse occurs in the majority of patients, and 70% eventually develop platinum resistance. For platinum‐resistant ovarian cancer (PROC), available non‐platinum chemotherapy options offer limited benefit, with poor efficacy (objective response rate [ORR] ≤ 15% and PFS ≤ 5 months). This unmet clinical need for PROC treatment has catalyzed exploration of ICIs. However, mono‐ICIs have generally produced disappointing results, as illustrated by KEYNOTE‐100 [55], UMIN000005714 [56], and NINJA [57], further underscoring that the well‐acknowledged immunosuppressive or immune‐excluded tumor microenvironment impedes ICI application in PROC.

A turning point emerged this year with trials testing combination strategies integrating immunotherapy with targeted and cytotoxic agents. In the phase III KEYNOTE‐B96/ENGOT‐ov60/GOG‐3063 trial, pembrolizumab plus weekly paclitaxel with or without bevacizumab improved OS compared with paclitaxel with or without bevacizumab in 643 PROC patients (18.2 vs. 14.0 months; *p* = 0.0053), supporting this triple combination as a new benchmark for this challenging subgroup [58]. As a step forward, the BRIGHT trial (Cohort 2) leveraged CD8+ tumor‐infiltrating lymphocyte (TIL) counts as a biomarker for ICI treatment in PROC [59]. In 72 BRCA1/2 wild‐type (BRCAwt) patients with ≥ 3 CD8+ TILs, a regimen of tislelizumab (anti‐PD‐1), bevacizumab, and nab‐paclitaxel achieved an ORR of 47.1%, median PFS of 7.3 months, and median OS of 18.4 months, superior to prior trials in unselected populations (median OS of 14.3 months) [58, 59]. In both studies, bevacizumab facilitates T‐cell trafficking by promoting vascular normalization [60], while nab‐paclitaxel/paclitaxel enhances antigen presentation thus create a pro‐inflammatory milieu [61]. Combined, these agents synergistically primed the tumor microenvironment to amplify ICI‐mediated T cell‐based anti‐tumor efficacy. Collectively, these findings establish a practical framework for precision immunotherapy, wherein ICIs achieve survival benefits in PROC by leveraging TME remodeling to overcome its “cold” immunosuppressive state.

Pathological subtype may also influence ICI efficacy. Ovarian clear cell carcinoma (OCCC), a rare subtype with limited therapeutic options, may uniquely benefit from immune‐based therapies. The multicenter, single‐arm phase II INOVA trial evaluated sintilimab (a PD‐1 inhibitor) plus bevacizumab in relapsed/refractory OCCC, reporting an ORR of 40.5% (95% CI 24.8%–57.9%), with a manageable safety profile. This combination highlights its potential for rare histological subtypes underserved by conventional treatments [62].

Collectively, although ovarian cancer is characterized as an “immune‐cold” malignancy with historically limited responsiveness to mono‐ICI therapy, emerging evidence suggests ICIs can achieve efficacy when strategically deployed precisely and personalized. The results of KEYNOTE‐B96 and BRIGHT support the value of rational combination strategies and biomarker‐guided patient selection, while recent findings in OCCC further suggest that certain histologic subtypes may derive particular benefit from immune‐based approaches. Together, these data highlight that the future of immunotherapy in ovarian cancer will depend on optimized combination, refined biomarkers, and more precise patient stratification.

#### 3.1.4 Precision Checkpoint Blockade

ICIs have become an important component of frontline treatment for selected gynecologic cancers, although their clinical benefit varies markedly by tumor type and molecular context. In cervical cancer, PD‐1/PD‐L1 blockade has moved into frontline treatment through combinations with chemoradiotherapy or chemotherapy. For endometrial cancer, robust responses in dMMR tumors underscore the critical role of molecular stratification, whereas pMMR tumors often require combination strategies—such as the addition of PARP inhibitors—to enhance tumor immunogenicity. By contrast, ovarian cancer, historically regarded as “immune‐cold,” exhibits limited efficacy with ICI monotherapy. Recent phase III trials, however, reveal that combination regimens designed to normalize tumor vasculature and activate anti‐tumor immunity can yield significant survival benefits in biomarker‐enriched subgroup. Collectively, these advancements highlight that the future of immunotherapy in gynecologic oncology hinges on two pillars: rigorous patient selection and precision multi‐agent integration. This evolving paradigm integrates tumor genomics, microenvironment modulation, and mechanism‐based treatment design to overcome resistance and move beyond a one‐size‐fits‐all approach to checkpoint blockade.

### 3.2 Advances in ADCs

**Figure 2:**
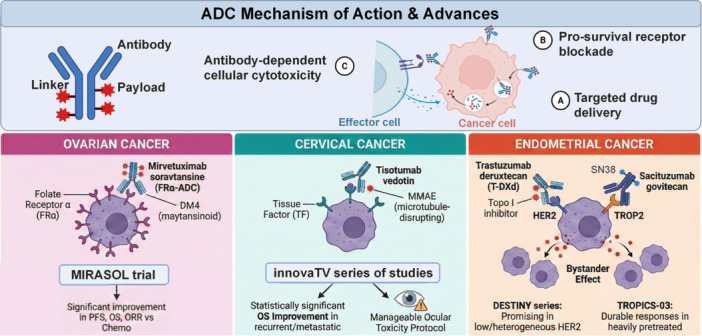
The structure of antibody‐drug conjugates, their mechanism against cancer and recent developments. Created in BioRender. Liu, J. (2026) https://BioRender.com/om7jsqa.

ADCs combine the precision of monoclonal antibodies with the powerful cytotoxic effects of small‐molecule payloads [63, 64]. By selectively delivering highly active cytotoxins to tumor cells expressing defined surface antigens, ADCs aim to maximize antitumor efficacy while minimizing systemic toxicity [65]. Advances in antibody engineering, linker stability, and payload chemistry have resulted in second‐ and third‐generation ADCs with improved pharmacokinetic profiles and therapeutic indices [66, 67]. This progress has sparked renewed clinical interest in ADCs for gynecologic cancers, particularly in settings marked by limited treatment options and high therapeutic resistance (Figure 2) [68, 69, 70, 71, 72].

#### 3.2.1 ADCs in Ovarian Cancer

Ovarian cancer is the most extensively studied gynecologic malignancy for ADCs development, primarily because tumor‐associated antigens show clinical relevant expression in this disease [73, 74, 75]. Among them, folate receptor alpha (FRα) is the most clinically validated target [76, 77]. Mirvetuximab soravtansine is an ADC directed against FRα and linked to the maytansinoid payload DM4 [78, 79, 80, 81, 82, 83]. In the phase III MIRASOL trial (NCT04209855), enrolling patients with FRα‐high, PROC, mirvetuximab soravtansine was compared with chemotherapy (paclitaxel, pegylated liposomal doxorubicin, or topotecan). The study demonstrated statistically significant and clinically meaningful improvements across all key endpoints, with a median PFS of approximately 5.6 versus 4.0 months for chemotherapy, and a median OS of 16.5 versus 12.8 months, corresponding to a clear reduction in the risk of disease progression and death. In addition, the ORR was markedly higher with mirvetuximab soravtansine (42%) compared with chemotherapy (16%) [84]. Importantly, MIRASOL is the first phase III ADC trial in ovarian cancer to demonstrate an OS benefit, providing definitive clinical validation of FRα as a therapeutic target and establishing biomarker‐selected ADC therapy as a successful development paradigm in platinum‐resistant disease, accompanied by better tolerability and fewer high‐grade systemic toxicities relative to chemotherapy. Collectively, MIRASOL redefined the standard for FRα‐high, PROC, elevated ADCs to practice‐changing therapies, and set a new benchmark for late‐phase drug development in ovarian cancer.

Additional ADC programs targeting NaPi2b, trophoblast cell surface antigen 2 (TROP2), CDH6, human epidermal growth factor receptor 2 (HER2), and mesothelin are in various early phases [85]. NaPi2b‐targeted ADC upifitamab rilsodotin has shown effectiveness in platinum‐resistant populations (ORR: 23% and DCR: 72%), while lifastuzumab vedotin has demonstrated responses in platinum‐sensitive recurrent populations (CA125 response: 54%) [86, 87]. CDH6‐targeted therapies have demonstrated promising response rates in human ovarian and kidney cancer models, supporting cadherin family members as viable therapeutic targets [88]. Currently, a phase I study (NCT04707248) is underway [89]. ADCs targeting TROP2 and mesothelin are also being investigated due to their broad tumor expression profiles, but evidence of durable survival benefits is still lacking [90, 91]. Many of these programs are in early stages, and challenges such as antigen expression variability, off‐target toxicity, and acquired resistance mechanisms remain significant issues.

Consequently, ADCs are beginning to assume a distinct therapeutic role in ovarian cancer, marking a transition from chemotherapy to targeted, antigen‐driven precision therapy.

#### 3.2.2 ADCs in Cervical Cancer

Unlike ovarian cancer, ADC development in cervical cancer has more recently focused on recurrent or metastatic disease [92]. Tissue factor (TF), a transmembrane glycoprotein involved in coagulation and tumor progression, is overexpressed in most cervical cancers and has been associated with aggressive tumor behavior [93, 94]. Tisotumab vedotin, an ADC composed of a TF‐directed monoclonal antibody linked to the microtubule‐disrupting agent monomethyl auristatin E (MMAE), has demonstrated significant antitumor activity in previously treated recurrent or metastatic cervical cancer [95]. Its clinical development has been evaluated across the innovaTV program, which includes early‐phase studies as well as the pivotal phase III innovaTV 301 trial [96, 97, 98, 99]. In innovaTV 301 (NCT04697628), a randomized, open‐label phase III trial in patients with recurrent or metastatic cervical cancer who had progressed after prior systemic therapy, tisotumab vedotin was compared with investigator's choice chemotherapy. Tisotumab vedotin significantly improved OS (11.5 vs. 9.5 months; HR = 0.70) and PFS (4.2 vs. 2.9 months, HR = 0.67), and was associated with a higher ORR (17.8% vs. 5.2%). These findings established TF‐targeted ADC therapy as an effective option in this heavily pretreated population [100]. While treatment is linked to specific toxicities, including ocular complications, these side effects are typically manageable and acceptable for advanced patients through appropriate preventive measures and multidisciplinary care [101]. The approval of tisotumab vedotin is a significant breakthrough, establishing ADCs as an effective treatment for advanced cervical cancer.

Additional targets are also being explored in cervical cancer. Nectin‐4 is of particular interest due to its frequent expression in tumors and minimal presence in normal tissues. Bulumtatug fuvedotin (BFv, 9MW2821), a next‐generation Nectin‐4 ADC, has demonstrated encouraging antitumor activity and manageable safety in a phase I/II study involving advanced solid tumors, including cervical cancer (ORR: 32.1%) [102]. HER2 and TROP2 have also been tested as potential targets in various basket trials [103, 104]. While phase III evidence is still lacking, the novel ADCs in advanced cervical cancer are evolving, supported by an expanding body of promising early‐phase clinical data.

#### 3.2.3 ADCs in Endometrial Cancer

In endometrial cancer, several ADC targets have been explored, particularly HER2 and TROP2 [105]. HER2 overexpression is particularly prevalent in uterine serous carcinoma, an aggressive histologic subtype with poor outcomes [106]. Trastuzumab deruxtecan, a HER2‐directed ADC carrying a topoisomerase I inhibitor payload [107, 108, 109], has shown promising efficacy in HER2‐positive solid tumors and has generated encouraging signals in endometrial cancer, including tumors with heterogeneous or low levels of HER2 expression in DESTINY‐PanTumor02 [103, 110]. Besides trastuzumab deruxtecan, HER2‐targeted ADC trastuzumab duocarmazine and trastuzumab emtansine were also tested in endometrial cancer as part of a trial [111, 112]. Overall, these findings indicate that ADCs may address some limitations of traditional HER2‐targeted therapies by delivering potent payloads and exhibiting bystander effects, although their use in endometrial cancer is not widespread.

Similarly, TROP2‐directed ADCs such as sacituzumab govitecan have shown encouraging results in early‐phase studies involving patients with heavily pretreated endometrial cancer [113, 114, 115]. The TROPiCS series trials investigate the efficacy and safety of treatments in heavily pretreated patients with various solid cancers, clarifying their role in Trop‐2‐expressing malignancies [90, 116, 117]. In TROPiCS‐03 (NCT03964727), a multicohort, open‐label Phase II study evaluates the TROP2‐directed ADC sacituzumab govitecan in advanced solid tumors, including a cohort for endometrial cancer. In heavily pretreated patients who had progressed after platinum‐based chemotherapy and often immune checkpoint blockade, the trial demonstrated an antitumor activity with an ORR of about 22%. These results indicate durable responses and manageable safety, supporting TROP2 as a viable therapeutic target for this challenging population [90].

#### 3.2.4 Targeted Delivery of Nonselective Cytotoxic Drugs

ADCs are revolutionizing systemic therapy for ovarian, cervical, and endometrial cancers by transitioning from nonselective chemotherapy to biomarker‐driven precision regimens. Notably, mirvetuximab soravtansine demonstrated improved OS for FRα‐high PROC. Tisotumab vedotin has shown a survival benefit in heavily pretreated cervical cancer. Meanwhile, a growing portfolio of ADCs—including those targeting NaPi2b, Nectin‐4, CDH6, mesothelin, and other tumor‐associated antigens, is under evaluation across multiple gynecologic malignancies. However, most remain in early‐phase (I/II) clinical development. Collectively, the efficacy of ADCs hinges on three pillars: rational antigen selection, biomarker‐guided patient stratification, and optimized treatment sequencing. Future advancements are anticipated to prioritize expanding validated targets, refining predictive biomarkers, integrating ADCs with immunotherapies/targeted agents, and elucidating resistance mechanisms to sustain therapeutic benefits.

## 4 Conclusion

In summary, recent clinical trials are reshaping the therapeutic landscape of gynecologic cancers, and accelerating the shift from traditional multimodal approaches toward more precise, evidence‐based management. In surgery, PHENIX supports nodal de‐escalation in selected early‐stage cervical cancer, whereas TRUST strengthens the case for PDS in fit patients with advanced ovarian cancer treated at expert centers. In systemic therapy, immunotherapy has achieved major gains through rational combinations and biomarker‐guided use, and ADCs have demonstrated meaningful efficacy in selected populations. However, challenges persist, including optimizing biomarker selection, managing overlapping toxicities, and addressing resistance mechanisms. Future high‐quality randomized controlled trials (RCTs) and large‐scale real‐world validation are essential to improve survival outcomes and redefine treatment standards in the field of gynecologic oncology.

## Author Contributions

**Yiyang Shen:** writing – review and editing, investigation, writing – original draft. **Xiaofei Jiao:** writing – review and editing, investigation, writing – original draft. **Yuanjia Wen:** investigation. **Zikun Peng:** investigation. **Jiahao Liu:** writing – review and editing, project administration, writing – original draft, supervision. **Xuejiao Zhao:** supervision, project administration, writing – review and editing. **Qinglei Gao:** supervision, project administration, writing – review and editing, writing – original draft.

## Ethics Statement

The authors have nothing to report.

## Consent

The authors have nothing to report.

## Conflicts of Interest

The authors declare no conflicts of interest.

## Data Availability

Data sharing is not applicable to this article as no datasets were generated or analyzed during the current study. The authors have nothing to report.
